# Malaria vectors and their blood-meal sources in an area of high bed net ownership in the western Kenya highlands

**DOI:** 10.1186/s12936-016-1115-y

**Published:** 2016-02-09

**Authors:** Bryson A. Ndenga, Nicholas L. Mulaya, Sandra K. Musaki, Joan N. Shiroko, Stefan Dongus, Ulrike Fillinger

**Affiliations:** Centre for Global Health Research, Kenya Medical Research Institute, P.O. 1578, Kisumu, 40100 Kenya; Vector Biology Department, Liverpool School of Tropical Medicine, Pembroke Place, Liverpool, L3 5QA UK; International Centre of Insect Physiology and Ecology, Thomas Odhiambo Campus, P.O. 30, Mbita, 40305 Kenya; Department of Disease Control, London School of Hygiene and Tropical Medicine, Keppel Street, London, WC1E 7HT UK

## Abstract

**Background:**

Blood-meal sources of malaria vectors affect their capacity to transmit the disease. Most efficient malaria vectors prefer human hosts. However, with increasing personal protection measures it becomes more difficult for them to find human hosts. Here recent malaria vector blood-meal sources in western Kenya highlands were investigated.

**Methods:**

Adult mosquitoes resting indoors, outdoors and exiting through windows were collected in three study areas within the western Kenya highlands from June 2011 to June 2013. A census of people, livestock and of insecticide-treated nets was done per house. Mosquito blood-meal sources were determined as human, goat, bovine or chicken using enzyme-linked-immunosorbent assays.

**Results:**

Most (86.3 %) households possessed at least one bed net, 57.2 % had domesticated animals and 83.6 % had people sharing houses with livestock at night. Most (94.9 %) unfed malaria vectors were caught exiting through windows. Overall, 53.1 % of *Anopheles gambiae* sensu stricto obtained blood-meals from humans, 26.5 % from goats and 18.4 % from bovines. Single blood-meal sources by *An. gambiae s.s.* from humans were 26.5 %, 8.2 % from bovines and 2.0 % from goats. Mixed blood-meal sources by *An. gambiae s.s.* identified included: 24.5 % human/goat, 10.2 % human/bovine, 8.2 % human/bovine/goat and also 8.2 % bovine/goat. One *An. arabiensis* mosquito obtained blood-meal only from humans.

**Conclusion:**

An unusually high frequency of animal and mixed human-animal blood meals in the major malaria vector *An. gambiae s.s.* was revealed in the western Kenya highlands where bed net coverage is above the WHO target. The shift in blood-meal sources from humans to livestock is most likely the vectors’ response to increased bed net coverage and the close location of livestock frequently in the same house as people at night. Livestock-targeted interventions should be considered under these circumstances to address residual malaria transmission.

## Background

Female mosquitoes take blood meals to obtain nutrients needed for egg development. In the process of obtaining blood meals from humans they can unwitting gain the potential to vector malaria parasites between hosts. The blood-meal source of malaria vectors affects the mosquito population’s capacity to transmit malaria, as illustrated by Garret-Jones’ model on vectorial capacity [1]. One of the factors included in the model is the frequency with which each mosquito bites a person, i.e., the probability that a particular mosquito will bite a human being rather than an animal on a given day.

Africa has the most efficient human malaria vectors, *Anopheles gambiae* sensu stricto and *An. funestus s.s.* and the most virulent malaria parasite, *Plasmodium falciparum* [2–5]. Their efficiency as malaria vectors is mainly due to their highly anthropophilic biting behaviour [2, 6–8]. These mosquitoes are the key malaria vectors in the western Kenya highlands [9, 10] where they have been shown in the past to prefer human blood meals and to feed indoors [6–8]. Similar feeding behaviours of malaria vectors were reported from the Kenyan coast about two decades ago [11]. These studies were done long before the introduction and scaling-up of insecticide-treated nets (ITNs)/long-lasting insecticidal nets (LLINs) and indoor residual spraying (IRS) to control malaria vectors.

Current malaria vector control methods are designed to prevent human-vector-contact and distribution of LLINs is aimed at universal coverage of all households in sub-Saharan Africa [12]. Insecticides on the net repels, disables and/kills mosquitoes that come into contact with them [13, 14]. The net also provides a physical barrier for mosquitoes to bite well-covered people. In areas where bed net coverage is high, malaria vectors might find it increasingly difficult to find a successful blood meal from their favourite host [10, 14, 15]. This study investigated malaria vector abundances and their blood-meal sources in an area of high bed net ownership in the western Kenya highlands.

## Methods

### Study area

**Fig. 1:**
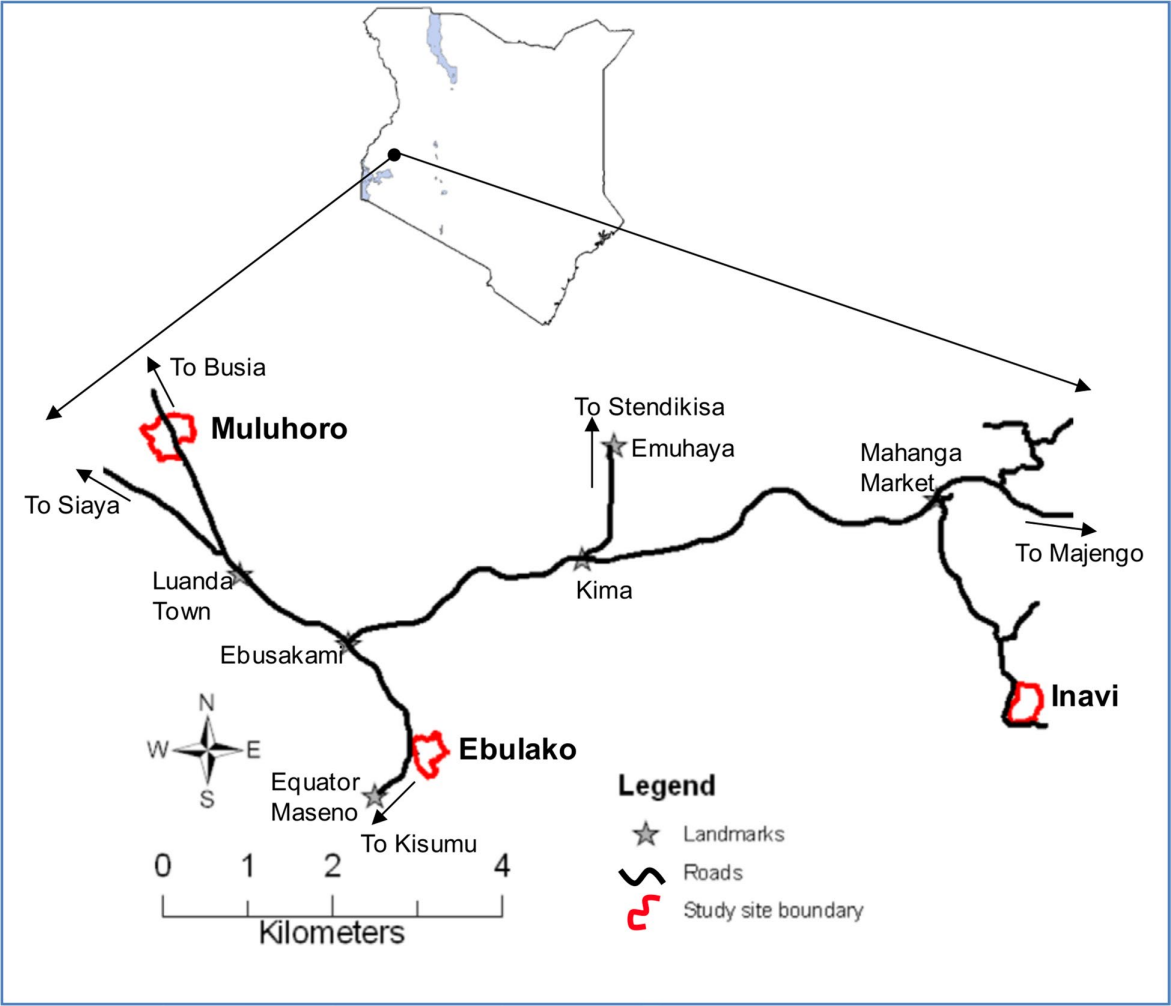
Map showing study areas (Ebulako, Muluhoro and Inavi) in Vihiga County within western Kenyan highlands

The study was conducted in three areas in Vihiga County from June 2011 to June 2013: Ebulako (latitude 0.006050, longitude 34.605891, 1534 m above sea level (asl), area 0.12 sq km); Muluhoro (latitude 0.037053, longitude 34.580801, l476 m asl, area 0.19 sq km); and, Inavi (latitude 0.008049, longitude 34.671638, 1658 m asl, area 0.12 sq km) (Fig. 1). These sites are characterized by undulating hills and valley bottoms with flowing streams, open drains and cultivated farms and homesteads on the slopes. Mosquito larval habitats have been associated with the agricultural activities in the valley bottoms where malaria vectors *An. gambiae s.s.*, *An. funestus s.s.*, *An. arabiensis*, *An. leesoni*, *An. rivulorum* and *An. vaneedeni* are found [16–18]. Malaria is a public health problem in Vihiga County with varying prevalence ranging from 20–65 % [16, 19]. Bed net ownership and usage in this area has been reported to range from 40–75 % [20]. The human population density was estimated to be 1045 persons per sq km according to the 2009 census [21]. Most houses are mud-walled with roofs of corrugated iron sheets, few houses are made of bricks or stones. Maize is the main crop in these sites, grown twice per year corresponding to the two annual rainy seasons from March to May and August to October. Livestock is kept on small-scale and includes cattle, goats, sheep, pigs, chickens, ducks, and quails [22].

### Mapping of houses

Study site boundaries were defined by walking on roads/footpaths and taking coordinates at several points surrounding each of the three sites: Ebulako, Muluhoro and Inavi which included a central valley bottom surrounded by residential houses on the slopes of the hills. Latitude, longitude and altitude of all houses within the study boundaries were mapped using a hand-held geographical positioning system (Garmin eTrex Legend^®^ HCx, Garmin International Inc, Olathe, Kansas, USA) at the beginning of the sampling period.

### Collection of adult mosquitoes

Mosquitoes resting indoors were collected using pyrethrum spray catches (PSC) [23] in all rooms of ten randomly selected sentinel houses, per site, twice per month for 25 months (June 2011–2013). Mosquitoes that exited through windows at night were collected daily using a window exit trap (WET) fitted in one of the windows [24] in ten houses per site for 23 months (August 2011 to June 2013). In an attempt to collect outdoor-resting mosquitoes, three resting pots (AgREPOTs) [25] were placed in each of the sites at a sentinel location approximately 5 m from a house under a banana canopy for 24 months (July 2011 to June 2013). From March 2012 to June 2013, one sticky resting box (SRB) was added at another sentinel location outside a house in each of the sites and sampled for 16 months.

### Household data

Two separate structured questionnaires were used to collect information on the mosquito control methods used and on livestock kept by the households in each of the three study areas. The household head or, if not available, any adult member of the house was the key respondent to these questionnaires. For mosquito control methods used, respondents were asked to state the number of household members ≤5 and >5 years old, the number of bed nets they owned, who slept under these nets and at what frequency and if any other mosquito control methods were used, including sprays, mosquito coils, topical repellents, screened windows, and larval control. This questionnaire was administered from 30 November, 2011 to 10 January, 2012. To explore the livestock-keeping habits, respondents were asked if they kept any animals, the type and the number they kept, where they slept at night and whether any person slept in the same house with the animals at night. This questionnaire was administered from 18–28 September, 2012.

### Mosquito identification

Mosquitoes were first sorted according to their generic groups, either as *Anopheles* or *Culex* males or females. Females were further sorted according to their gonotrophic status as unfed, blood-fed, half-gravid or gravid. The *Anopheles* mosquitoes were further identified as either *An. gambiae s.l.* or *An. funestus s.l.* using morphological features [26, 27]. Individual specimens of female *An. gambiae s.l.* were further identified to species either as *An. gambiae s.s.* or *An. arabiensis* using the rDNA-polymerase chain reaction (PCR) method [28]. Female blood-fed *An. gambiae s.l.* mosquitoes tested for blood-meal sources were also individually identified by PCR as either *An. gambiae s.s.* or *An. arabiensis*.

### Blood-meal source identification

Blood-fed *An. gambiae s.s.* and *An. arabiensis* mosquitoes were individually processed and analysed to determine antihost Immunoglobulin G (IgG) conjugate against human, goat, bovine, and chicken using a direct enzyme-linked-immunosorbent assay (ELISA) as described by Beier et al. [7]. The results were read at 414 nm wavelength (Multiskan Ex Lab Systems Type-355, Helsinki, Finland).

### Antibodies

Antibodies used to test for human blood were goat anti-human, IgG heavy + light chains (H + L), liquid, horseradish peroxidase (HRP), product code 474-1006 and goat anti-human, IgG (H + L) product code 01-10-06. Those used to test for bovine blood were goat anti-bovine, IgG (H + L), HRP, product code 14-12-06 and goat anti-bovine, IgG (H + L), product code 01-12-06. Those used to test for goat blood were rabbit anti-goat, IgG (H + L), HRP, product code 14-13-06 and rabbit anti-goat, IgG (H + L), product code 01-13-06. The antibodies used to test for chicken blood were goat anti-chicken, IgG (H + L), HRP, product code 14-24-06 and goat anti-chicken, IgG (H + L), product code 01-24-06. All these antibodies were obtained from Kirkegaard and Perry Laboratories Inc, Gaithersburg, MD, USA.

### Ethical considerations

After the identification and selection of the three study areas (Ebulako, Muluhoro and Inavi), meetings with local government administration officers (chiefs and their assistant chiefs) were held in each of these areas. Chiefs’ public meetings, locally known as *barazas*, with the local residents were organized and attended on 20 May, 2011, 8 June, 2011 and 21 July, 2011 for Muluhoro, Ebulako and Inavi sites, respectively. In these meetings, the objectives of the study, methods and duration were discussed and study staff were introduced to the public. Before any sampling was initiated, written and signed consent was obtained from each household head in order to enrol his/her house on the study and access their private land. Consent forms were translated to local languages and used as per the respondents’ choice. A copy of the signed consent form was given to the person who signed it and another kept in a cabinet with restricted access in the office at Kenya Medical Research Institute, Centre for Global Health Research station at Kisian in Kisumu. Permissions were required to access all locations and private premises in all the three study areas. No endangered or protected species were involved in this study. This study was approved by KEMRI/National Ethical Review Committee (SSC No. 1963).

### Data analysis

Data collected included number of bed nets, number of residents and domesticated animals, number of adult mosquitoes and their gonotrophic stages. Blood-meal sources were expressed as counts and proportions. Count data from the questionnaires, e.g., number of residents, bed nets and domesticated animals among the three study areas were analysed using one-way analysis of variance (ANOVA) and Tukey–Kramer honestly significant difference (HSD) tests were used to compare the means among the three sites in SPSS version 20.0. Analyses to determine differences in the densities of blood-fed malaria vectors collected by PSC and WET among the three study areas (Ebulako, Muluhoro and Inavi) were performed using generalized estimating equations (GEE) on count data that were fitted with a negative binomial distribution with a log link function. Differences in the monthly mean density for each of the adult mosquito collection methods (PSC, WET, AgREPOTs, SRB) were also compared using GEE. House/trap identity was treated as the subject variable and an exchangeable correlation matrix chosen for the repeated measurements (the total number of mosquitoes collected per house/trap). Since collection of adult mosquitoes using these methods started at different times, data collected in the same period, from March 2012 to June 2013, were used in this analysis.

## Results

A total of 193 residential houses were mapped within the boundaries of the three study areas but household data on mosquito control methods could only be obtained from 153 houses and data on domesticated animals from 152 houses (Table 1). This was due to absence of the residents of the house on the survey dates or refusal to consent to be enumerated. A total of 568 persons were counted in the 153 houses, with an average of 3.71 (3.41–4.02) persons/household (Table 1). Little less than a fifth (18.3 %) of the population was under the age of 5 years. The three study sites were similar in their mean number of household members and in the mean number of domesticated animals kept per household. The only difference between sites was bed net ownership which was highest in Ebulako with a mean of 2.13 nets per household and lowest in Inavi with a mean of 1.43 nets per household (Table 1).

**Table 1:** Mean density of people, bed nets and domesticated animals per house within the three study sites

	Ebulako	Muluhoro	Inavi	Total
Houses per site	70	60	63	193
Houses sampled 1	53 (75.7)	49 (81.7)	51 (81.0)	153 (79.3)
Persons	3.68 (3.18–4.18)^a^	4.16 (3.53–4.80)^a^	3.31 (2.86–3.77)^a^	3.71 (3.41–4.02)
Bed nets	2.13 (1.80–2.46)^a^	1.67 (1.27–2.08)^a,b^	1.43 (1.14–1.72)^b^	1.75 (1.55–1.95)
Houses sampled 2	23 (32.9)	70 (116.7)	59 (93.7)	152 (78.8)
Cats	0.22 (0.04–0.40)^a^	0.16 (0.07–0.24)^a^	0.14 (0.05–0.23)^a^	0.16 (0.10–0.22)
Chickens	2.09 (0.55–3.62)^a^	2.81 (1.59–4.04)^a^	4.81 (2.80–6.82)^a^	3.48 (2.49–4.47)
Cows	1.09 (0.31–1.87)^a^	0.73 (0.44–1.02)^a^	1.37 (0.88–1.87)^a^	1.03 (0.77–1.29)
Dogs	0.17 (−0.19–0.53)^a^	0.04 (−0.02–0.11)^a^	0.19 (0.02–0.35)^a^	0.12 (0.03–0.20)
Goats	0.43 (−0.13–1.00)^a^	0.23 (0.05–0.41)^a^	0.32 (0.09–0.56)^a^	0.30 (0.15–0.44)
Sheep	0	0.13 (−0.01–0.27)^a^	0.05 (−0.05–0.15)^a^	0.08 (0.00–0.15)

Numbers in parentheses are percentages for houses sampled and are 95 % CI for the others

Letters following numbers indicate the results of Tukey–Kramer HSD tests. Values with the same letter in a row were not statistically significant at P < 0.05

Out of the 153 households that were sampled, 132 (86.3 %) had bed nets with a total of 511 residents. Twenty-one (13.7 %) households were without bed nets and had a total of 57 residents. Fifty (32.7 %) households had one bed net each and with a total of 132 residents. Forty-four (28.8 %) households had two bed nets each and a total of 186 residents. Twenty-nine (19.0 %) households had three bed nets each and a total of 140 residents. Six (3.9 %) households had four bed nets each and a total of 32 residents. Only one (0.7 %) household had five bed nets with four residents. Two (1.3 %) households had seven bed nets each and with a total of 17 residents. On average, one bed net was used by 2.1 (568/268) people. Bed nets were the main malaria prevention method used in the communities. These were supplemented by anti-mosquito sprays in 5.2 % (8/153) of the households and mosquito coils in 2.0 % (3/153). No other mosquito control measures were used within the three sites.

Over a half (57.2 %, 87/152) of the interviewed households had domesticated animals. A total of 785 animals were kept, most of them being chickens (Table 2). Most of the domesticated animals slept in the main house (86.0 %), few in the sheds (5.7 %), in the kitchen (5.2 %), and outside (3.1 %) in the open (Table 2). The majority of households (83.6 %, 158/189) had people and domesticated animals sharing the same houses, including kitchens, at night. It is especially notable that the majority of cattle, goats and chickens were kept in the main house during the night (Table 2).

**Table 2:** Number (percentage) of domesticated animals and where they slept at night

Type	Main house	Kitchen	Shed	Outside	Total (%)
Chickens	496 (93.8)	17 (3.2)	16 (3.0)	0	529 (67.4)
Cows	108 (68.8)	22 (14.0)	22 (14.0)	5 (3.2)	157 (20.0)
Goats	37 (82.2)	2 (4.4)	6 (13.3)	0	45 (5.7)
Cats	22 (91.7)	0	1 (4.2)	1 (4.2)	24 (3.1)
Dogs	0	0	0	18 (100)	18 (2.3)
Sheep	12 (100)	0	0	0	12 (1.5)
Total (%)	675 (86.0)	41 (5.2)	45 (5.7)	24 (3.1)	785

A total of 80,331 mosquitoes were collected: 76.5 % (61,459/80,331) of them were collected exiting through windows at night; 9.9 % (7943/80,331) were collected resting indoors; 7.4 % (5919/80,331) resting outdoors in AgREPOTs and 6.2 % (5010/80,331) resting outdoors in SRB (Table 3). A total of 1911 malaria vectors were unfed and most of them (94.9 %, 1814/1911) were caught exiting through the window traps. The majority (94.6 %) (76,033/80,331) of the mosquitoes were *Culex* species, 4.3 % (3493/80,331) were *An. gambiae s.l.* and 1.0 % (805/80,331) was *An. funestus s.l.*. Most of the females caught were unblood-fed: 80.6 % (1602/1987) of the collected *An. gambiae s.l.*; 72.8 % (398/546) of the *An. funestus s.l.* and 47.2 % (23,689/50,205) of the *Culex* species. The overall mean density of *An. gambiae s.l.* in PSC was 0.17 (95 % confidence interval (CI) 0.13–0.22) and 0.04 (95 % CI 0.03–0.06) in WET. The *An. funestus s.l.* overall mean density was 0.02 (95 % CI 0.01–0.04) in PSC and 0.01 (95 % CI 0.01–0.02) in WET. Means of *An. gambiae s.l.* and *An. funestus s.l.* in AgREPOTs and SRB could not be modelled due to the low numbers that were collected outdoors. The mean density of *Culex* spp. was 3.06 (95 % CI 2.75–3.39) in PSC, 2.79 (95 % CI 1.93–4.03) in WET, 0.85 (95 % CI 0.74–0.97) in AgREPOTs, and 3.14 (95 % CI 3.14–3.14) in SRB. Average monthly numbers of adult mosquitoes collected in WET, AgREPOT and SRB were significantly lower than those collected by PSC, except for *An. funestus s.l.* in WET which was not significantly different (Table 4).

**Table 3:** Number of adult mosquitoes and their gonotrophic stages collected using different methods

Gonotrophic stages	PSC	WET	AgREPOTs	SRB	Total (%)
*Anopheles gambiae*					
Males	142	1416	2	1	1561 (44.7)
Unfed	80	1475	1	1	1557 (44.6)
Blood-fed	86	120	0	2	208 (6.0)
Half-gravid	26	28	0	0	54 (1.5)
Gravid	41	71	1	0	113 (3.2)
Total	375	3110	4	4	3493 (4.3)
*Anopheles funestus*					
Males	27	288	3	1	319 (39.6)
Unfed	8	339	3	4	354 (44.0)
Blood-fed	17	37	2	0	56 (7.0)
Half-gravid	12	8	2	1	23 (2.9)
Gravid	7	37	9	0	53 (6.6)
Total	71	709	19	6	805 (1.0)
*Culex* species					
Males	2979	18,224	2754	1937	25,894 (34.1)
Unfed	1734	19,030	1558	1336	23,658 (31.1)
Blood-fed	933	4479	172	140	5724 (7.5)
Half-gravid	957	7760	672	771	10,160 (13.4)
Gravid	894	8147	740	816	10,597 (13.9)
Total	7497	57,640	5896	5000	76,033 (94.6)
Grand total (%)	7943 (9.9)	61,459 (76.5)	5919 (7.4)	5010 (6.2)	80,331

*PSC* pyrethrum spray catches; *WET* window exit traps; *AgREPOTs* *Anopheles gambiae* resting pots; *SRB* sticky resting boxes

**Table 4:** Statistical comparison of the monthly mean densities per trap of adult mosquitoes collected using the pyrethrum spray catches, window exit traps, *Anopheles gambiae* resting pots and sticky resting boxes

Mosquito type	Collection method	Odds ratio (95 % CI)	P
*An. gambiae s.l.*	SRB	0.026 (0.018–0.038)	<0.001
	AgREPOT	0.004 (0.002–0.008)	<0.001
	WET	0.486 (0.369–0.638)	<0.001
	PSC	1.000	
*An. funestus s.l.*	SRB	0.179 (0.061–0.523)	0.002
	AgREPOT	0.143 (0.051–0.397)	<0.001
	WET	0.798 (0.550–1.156)	0.233
	PSC	1.000	
*Culex* spp.	SRB	0.640 (0.478–0.857)	0.003
	AgREPOT	0.156 (0.104–0.232)	<0.001
	WET	0.639 (0.530–0.771)	<0.001
	PSC	1.000	

*PSC* pyrethrum spray catches; *WET* window exit traps; *AgREPOTs* *Anopheles gambiae* resting pots; *SRB* sticky resting boxes

The mean density of blood-fed malaria vectors per house was low in the study areas in general, however, significant differences were observed between the study areas (Table 5). It was 8.2–9.4 times more likely to collect a blood-fed *An. gambiae s.l.* in Muluhoro than in the two other study sites and 9.6 times more likely to collect a blood-fed *An. funestus s.l.* (Table 5). Out of the 208 blood-fed adult *An. gambiae s.l.* mosquitoes collected (Table 3), 23.6 % (49) were analysed by ELISA to determine the sources of their blood meals and also by PCR to determine their species.

**Table 5:** Mean density of blood-fed malaria vectors collected in Muluhoro, Ebulako and Inavi

Collection method	Species	Site	Site mean (95 % CI)	Odds ratio (95 % CI)	P
PSC	*An. gambiae s.l.*	Inavi	0.014 (0.007–0.028)	0.112 (0.039–0.323)	<0.001
		Ebulako	0.034 (0.013–0.089)	0.276 (0.079–0.967)	0.044
		Muluhoro	0.124 (0.055–0.276)	1.000	
PSC	*An. funestus s.l.*	Inavi	0.002 (0.000–0.013)	0.064 (0.009–0.474)	0.007
		Ebulako	0.002 (0.000–0.013)	0.064 (0.009–0.474)	0.007
		Muluhoro	0.031 (0.015–0.064)	1.000	
WET	*An. gambiae s.l.*	Inavi	0.001 (0.000–0.001)	0.036 (0.011–0.113)	<0.001
		Ebulako	0.001 (0.000–0.002)	0.045 (0.014–0.148)	<0.001
		Muluhoro	0.017 (0.007–0.039)	1.000	

Blood-meals sources identified from *An. gambiae s.s.* were 53.1 % from humans, 26.5 % from goats and 18.4 % from bovines (Table 6). Some *An. gambiae s.s.* mosquitoes obtained blood-meals from single sources, that is, 26.5 % from humans, 8.2 % from bovines, 2.0 % from goats. One (2.0 %) *An. arabiensis* obtained blood from humans only (Table 6). No blood-meal from chickens was identified from both *An. gambiae s.s.* and *An. arabiensis*. Mixed blood-meal sources identified from *An. gambiae s.s.* included: 24.5 % (12/49) human/goat, 10.2 % (five) human/bovine, 8.2 % (four) human/bovine/goat and 8.2 % (four) bovine/goat (Table 6). A total of 564 *An. gambiae s.l.* were further identified by PCR: 383 (67.9 %) were *An. gambiae s.s.*, four (0.7 %) were *An. arabiensis* and 177 (31.4 %) were not identified due to amplification failure.

**Table 6:** Verified blood-meal sources for *Anopheles* *gambiae s.s.* and *Anopheles* *arabiensis*

Species	*Anopheles gambiae s.s.*	*Anopheles arabiensis*
	Proportion % (count/49)	Proportion % (count/49)
Blood-meal sources		
Human	53.1 (26)	2.0 (1)
Goat	26.5 (13)	0.0 (0)
Bovine	18.4 (9)	0.0 (0)
Single blood-meal sources		
Human	26.5 (13)	2.0 (1)
Bovine	8.2 (4)	0.0 (0)
Goat	2.0 (1)	0.0 (0)
Mixed blood-meal sources		
Human/goat	24.5 (12)	0.0 (0)
Human/bovine	10.2 (5)	0.0 (0)
Human/bovine/goat	8.2 (4)	0.0 (0)
Bovine/goat	8.2 (4)	0.0 (0)

## Discussion

There has been a dramatic increase in the proportion of households owning at least one bed net, from 3 % in 2004 to estimated 49 % (range 44–54 %) in 2013 in sub-Saharan Africa (WHO 2014). In Kenya, LLIN ownership was estimated in 2008 to be 56 % nationwide [29] and more recent reports from western Kenya show ownership and usage to exceed the World Health Organization (WHO) target of 80 % coverage [10, 30, 31]. The latter can be confirmed by the here presented data. In the study area in the western Kenya highlands bed net coverage was 86 % and the recommended minimum bed net coverage ratio of one LLIN per two persons at risk of malaria [32] has been achieved. However, this achievement may be distorted by the existence of a gap between bed net coverage and usage, which was not further explored in this study but which has been shown to vary among sites in the area and seasons with greater usage during high malaria transmission seasons [20].

The physical barrier preventing a blood meal combined with a repellent effect of the LLINs may have led fewer malaria vectors to rest, feed and remain indoors until they became gravid. This is in agreement with other studies that reported a reduced likelihood of malaria vectors to obtain blood meals indoors but an increased likelihood to exit from houses with bed nets [33, 34]. Outdoor mosquito collection tools used in this study, namely AgREPOTs and SRBs however, failed to sample the mosquitoes outdoors. Hence, the blood-meal sources results presented in this study has the bias of only using mosquitoes collected indoors. Therefore, there is an urgent need to investigate where malaria vectors rest outdoors after leaving the houses in this study area in order to develop more efficient sampling tools.

Animal husbandry is common and a key economic activity within the western Kenya highlands [22]. Livestock are mainly kept for food, sale of their products and as a financial security in times of need including the payment of pride prices. Dogs are kept to offer security at night and cats to keep rats and snakes away from the house. Due to increased human population density [21] and livestock theft cases [22]; the practice of most households sharing the same houses at night with livestock is common in these areas. This is done to enhance livestock’s security at night. This puts both people and domesticated animals in close vicinity to malaria vectors at night.

An interaction between increased bed net coverage of humans and the presence of domesticated animals in the same houses at night likely plays a key role in this increased observation of animal and mixed human-animal blood meals taken by local malaria vectors. This is likely enhanced by the physical, chemical (excito-repellence, especially pyrethroid insecticides) and community-wide barriers that LLINs offer humans against malaria vectors [14]. Malaria vectors that fail to obtain blood meals from humans are compelled to seek and obtain from other readily available animal sources. A similar diversion of *An. gambiae s.s.* and *An. arabiensis* mosquitoes from feeding on humans to cattle was reported about three and half decades ago and was attributed to insecticidal spraying using fenitrothion [35].

Blood-meal sources were tested only for origin from humans, goats, bovines, and chicken, but there is a possibility that malaria vectors obtain blood meals from all available domesticated animals, as shown by a large number of previous and recent studies indicating that the major malaria vectors in sub-Saharan Africa readily adapt to available blood-meal hosts even if they have a preference for human hosts [6, 7, 11, 36–45]. Approximately two decades ago, the human blood index (HBI) for *An. gambiae s.s.* collected indoors within the region north of the Lake Victoria (Kisian, Saradidi and Mumias) was 88–97 % an indication that they had fed exclusively on humans [6–8]. However, in this study, the HBI was 53 % and, may be more importantly, the majority of those that had fed on humans had also fed on animals. This strongly suggests a shift in blood-meal sources as a result of the interaction between increased bed net coverage and close proximity of domesticated animals. Comparing the data from this study with the data from Shililu et al. collected in this region two decades ago before bed nets were used in large numbers and treated with insecticides [8, 46], blood-meal sources from humans have dropped by 43 % (96 % minus 53 %), and from bovines has increased by 13 % (5 % minus 18 %). Mutuku et al. [39] reported a similar reduction in HBI and increased blood-meal sources from cattle and goats following increased ITN use on Kenya’s south coast. The key implication of this shift of malaria vector blood-meal sources from humans to domesticated animals is a reduction in malaria transmission as the *Plasmodium* parasites that cause human malaria do not develop fully in the domesticated animals. Infective stages of the malaria parasite (sporozoites) injected in animals by malaria vectors, in the process of taking blood meals, reach a dead end in their development cycle. This is enhanced by the fact that malaria vectors obtain blood meals from multiple hosts, including different types of domesticated animals. This may have contributed to the decline in malaria epidemics, prevalence, incidence, and distribution that is being reported in Kenya [47].

A shift of malaria vector blood-meal sources from humans to animals presents an opportunity for their control, especially in areas where livestock is kept in close vicinity to people. For example, insecticides can be applied on livestock in order to kill the malaria vectors when biting [48–50]. However, the few relevant studies conducted to date have focused on pyrethroid-treated cattle [48, 49]. The insecticides tested are identical to those used for mosquito control indoors and subject to increasing levels of resistance [51]. Accordingly, treating cattle with pyrethroids is unlikely to be sustainable and would contribute to even more rapid development of resistance. Furthermore, it has been shown, with few exceptions, that the impact of pyrethroids on cattle is effective for less than 1 week [52–54] which would require weekly re-application. In addition, some pyrethroids have shown repellent effects on mosquitoes [55] which can be highly counterproductive, as vectors could be diverted to feed on people, thereby increasing transmission [56]. Consequently, there is a need to develop cattle-targeted interventions based on insecticides with a completely different mode of action to insecticides currently used (i.e., pyrethroids, organophosphates) or proposed (i.e., chlorfenapyr) to control mosquitoes indoors. Promising novel control agents might be cattle endectocides or insect growth regulators [57]. It has been suggested that treatment of livestock in the entire community at the same time can maximize protection from malaria vectors and other livestock ecto-parasites and biting flies [58]. Such an approach is likely to get community support due to its added advantage of protecting both humans and livestock. Zooprophylaxis and the use of insecticide-treated cattle have been thought to work well for *An. arabiensis*, which shows naturally a more zoophilic behaviour [59, 60] but has rarely been suggested for other malaria vectors. However, the findings of this study suggest that it can also work for *An. gambiae s.s*., in areas where humans are well covered by bed nets and where malaria vectors, humans and livestock are at close vicinity at night.

## Conclusion

This study revealed an unusually high frequency of animal and mixed human-animal blood meals in the major malaria vector, *An. gambiae s.s.,* in the western Kenya highlands where, at the same time, the average number of malaria vectors are low (between 0.01 and 0.17 per trap night) and where LLIN coverage of people is above the WHO target. The shift in blood-meal sources from humans to livestock is most likely the vectors’ response to increased LLIN coverage and the close location of livestock, frequently in the same house as people at night. Frequent blood meals of malaria vectors from livestock hosts present a novel opportunity to control the residual transmission with livestock-targeted interventions.
